# Late Changes in the Extracellular Matrix of the Bladder after Radiation Therapy for Pelvic Tumors

**DOI:** 10.3390/diagnostics11091615

**Published:** 2021-09-04

**Authors:** Olga Streltsova, Elena Kiseleva, Varvara Dudenkova, Ekaterina Sergeeva, Ekaterina Tararova, Marina Kochueva, Svetlana Kotova, Victoriya Timofeeva, Katerina Yunusova, Anna Bavrina, Peter Timashev, Anna Solovieva, Anna Maslennikova

**Affiliations:** 1 E.V. Shakhov Department of Urology, Privolzhsky Research Medical University, 10/1 Minin and Pozharsky Sq., 603950 Nizhny Novgorod, Russia; strelzova_uro@mail.ru; 2 Institute of Experimental Oncology and Biomedical Technologies, Privolzhsky Research Medical University, 10/1 Minin and Pozharsky Sq., 603950 Nizhny Novgorod, Russia; orannge@mail.ru (V.D.); marina.kochueva@mail.ru (M.K.); 3 Institute of Applied Physics of the RAS, 46 Ulyanova St., 603950 Nizhny Novgorod, Russia; sea@ufp.appl.sci-nnov.ru; 4 Nizhny Novgorod Regional Oncology Dispensary, 190 Rodionova St., 603126 Nizhny Novgorod, Russia; tararova-ea@mail.ru; 5 Department of Polymers and Composites, N.N. Semenov Federal Research Center for Chemical Physics, 4 Kosygin St., 119991 Moscow, Russia; slkotova@mail.ru (S.K.); vik.timofeeva@gmail.com (V.T.); timashev.peter@gmail.com (P.T.); ann.solovieva@gmail.com (A.S.); 6 Institute for Regenerative Medicine, I.M. Sechenov First Moscow State Medical University, 8 Trubetskaya St., 119991 Moscow, Russia; 7 The Department of Anatomical Pathology, Privolzhsky Research Medical University, 10/1 Minin and Pozharsky Sq., 603950 Nizhny Novgorod, Russia; katyayunusova@ya.ru; 8 Department of Medical Physics and Informatics, Privolzhsky Research Medical University, 10/1 Minin and Pozharsky Sq., 603950 Nizhny Novgorod, Russia; annabavr@rambler.ru; 9 Chemistry Department, Lomonosov Moscow State University, Leninskiye Gory 1-3, 119991 Moscow, Russia; 10 Department of Oncology, Radiation Therapy and Radiological Diagnostics, Privolzhsky Research Medical University, 10/1 Minin and Pozharsky Sq., 603950 Nizhny Novgorod, Russia; maslennikova.anna@gmail.com; 11 Department of Biophysics of Institute of Biology and Biomedicine, Lobachevsky State University of Nizhny Novgorod, 23 Gagarin Avenue, BLDG 2, 603022 Nizhny Novgorod, Russia

**Keywords:** radiation-induced damage, extracellular matrix, cross-polarization optical coherence tomography, two-photon microscopy, atomic force microscopy, image analysis

## Abstract

Radiation therapy is one of the cardinal approaches in the treatment of malignant tumors of the pelvis. It leads to the development of radiation-induced complications in the normal tissues. Thus, the evaluation of radiation-induced changes in the extracellular matrix of the normal tissue is deemed urgent, since connective tissue stroma degradation plays a crucial role in the development of Grade 3–4 adverse effects (hemorrhage, necrosis, and fistula). Such adverse effects not only drastically reduce the patients’ quality of life but can also become life-threatening. The aim of this study is to quantitatively analyze the bladder collagen state in patients who underwent radiation therapy for cervical and endometrial cancer and in patients with chronic bacterial cystitis and compare them to the normal bladder extracellular matrix. Materials and methods: One hundred and five patients with Grade 2–4 of radiation cystitis, 67 patients with bacterial chronic cystitis, and 20 volunteers without bladder pathology were enrolled. Collagen changes were evaluated depending on its hierarchical level: fibrils and fibers level by atomic force microscopy; fibers and bundles level by two-photon microscopy in the second harmonic generation (SHG) mode; general collagen architectonics by cross-polarization optical coherence tomography (CP OCT). Results: The main sign of the radiation-induced damage of collagen fibrils and fibers was the loss of the ordered “basket-weave” packing and a significant increase in the total area of ruptures deeper than 1 µm compared to the intact sample. The numerical analysis of SHG images detected that a decrease in the SHG signal intensity of collagen is correlated with the increase in the grade of radiation cystitis. The OCT signal brightness in cross-polarization images demonstrated a gradual decrease compared to the intact bladder depending on the grade of the adverse event. Conclusions: The observed correspondence between the extracellular matrix changes at the microscopic level and at the level of the general organ architectonics allows for the consideration of CP OCT as a method of “optical biopsy” in the grading of radiation-induced collagen damage.

## 1. Introduction

Radiation therapy (RT) is one of the basic approaches in the treatment of malignant tumors of the pelvis (i.e., cervical cancer, endometrial cancer, prostate cancer). Irradiation for these neoplasms assumes a combination of an external-beam therapy and high-dose-rate brachytherapy that leads to the development of radiation-induced complications in normal tissues [1,2]. The degree of their severity may vary from mild functional abnormalities to a damage significantly affecting the patient’s quality of life [3]. Earlier, in the era of conventional irradiation, the incidence of the severe (Grade > 2) adverse effects of radiation therapy of pelvic organs ranged between 15% and 30% [4,5,6]. During the last 20 years, intensity-modulated RT techniques have been developed, which employ a high dose gradient at the target/normal tissue border. Their application allowed an essential reduction in the incidence and severity of RT complications affecting the rectum and bladder [7,8,9]. However, it has not managed to eliminate the problem of RT adverse effects completely.

The pathogenesis of radiation-induced lesions of normal tissues is complicated and involves interaction of a large number of tissue and cellular factors [10], development of a vascular reaction [11], as well as progressive changes of the extracellular matrix (ECM) [12,13]. Progressive changes in ECM is of major importance, since connective tissue stroma degradation plays a crucial role in the development of Grade 3–4 adverse effects (hemorrhage, necrosis, and fistula), which drastically reduce the patients’ quality of life and provoke life-threatening conditions [3].

Thus, the task of evaluation of radiation-induced changes in the ECM of the normal tissue, at various levels of its spatial arrangement, is crucial. The optical and physical diagnostic techniques developed over the last two decades have unlocked new opportunities for a detailed study of extracellular stromal structures at the level of fibrils and fibers (atomic force microscopy, AFM), fibers and their bundles (two-photon microscopy, TPM), and general tissue architecture (optical coherence tomography, OCT), both ex vivo [14,15] and in vivo [16,17]. In particular, AFM, which has been an instrumental tool in the biomedical studies [18], is currently widely used for the observation of the ECM complex architecture and mechanical properties at the micro- and nanoscale [19,20,21,22,23].

In vivo studies of the collagen state after application of ionizing radiation in clinically relevant doses in tendon collagen [24] and in an animal model [15] by optical and physical techniques allowed for the revealing of the basic principles of the development of collagen radiation destruction and subsequent remodeling, depending on the level of its hierarchical organization (molecules, fibrils, fibers, and fiber bundles). The main manifestations of the post-radiation collagen alterations at the level of fibrils and fibers (at the nanoscale) appeared to be the formation of thicker fibers relative to the intact ones, disordering of the 3D structure and formation of deep ruptures in the matrix structure, as well as appearance of a large amount of an amorphous substance (presumably, proteoglycans) [25]. TPM revealed a decrease in the intensity of the second harmonics generation (SHG) signal from collagen [15] that reflected the processes of degradation and disordering of its 3D structure. At the level of general architecture of the bladder tissue, the main manifestation of collagen radiation damage was the degradation/fragmentation of collagen fibers. It has been revealed in vivo by cross-polarization (CP) OCT as the diminished signal intensity from the submucosa in the cross-polarization channel, which is sensitive to collagen fibers organization (their anisotropy) [15]. These findings were verified with histological evaluation.

The results of experimental studies became a basis for the transfer of the methods into the clinic. TPM in the SHG mode allowed for the obtaining of the qualitative characteristics of the ECM state for adverse effects of various degrees of severity [26,27] and their quantitative estimation [28]. This study is an extension and enhancement of our previous studies and is dedicated to a complex evaluation of the bladder ECM state at various levels of its hierarchical arrangement, after irradiation for pelvic malignant tumors. We compare the observed bladder changes related to radiation cystitis and the changes in the bladder for chronic cystitis, as well as explore the possibilities of CP OCT as a technique for non-invasive diagnostics of the severity of the bladder radiation damage.

## 2. Materials and Methods

### 2.1. The Patients’ Data and the Study Design

CP OCT study of the bladder mucosa state was conducted in 105 female patients who earlier (from 2 to 15 years) had received RT for cervical and endometrial cancer, in 67 female patients who had received treatment for 3–5 years for bacterial chronic cystitis, and in 20 volunteers with no known bladder pathology. The study was approved by the Ethical Committee of the Privolzhsky Research Medical University (Protocol No.14 from 10 December 2013). All the patients had signed the informed consent for participation in the study. From 9 patients of the radiation cystitis group and from 12 patients of the chronic cystitis group, a biopsy material was taken, which was later studied by TPM in the SHG mode and AFM. To examine the intact bladder by TPM and AFM, 5 samples of autopsy-derived material were obtained with the same age and gender characteristics, without the genitourinary system pathology. The generalized data of the patients included in the study are presented in Table 1.

The study included the following procedures:

1. Cystoscopy of the bladder in all the patients with a visual assessment of the mucosa according to the protocol.
2. CP OCT study of the bladder mucosa. For each patient, at least 2–3 images were acquired for the bladder bottom region—the “hot” zone receiving the highest radiation dose in pelvic tumor RT.
3. Biopsy harvesting. Biopsy was performed with a resectoscope (Karl Storz SE and Co. KG, Tuttlingen, Germany) from the regions of the CP OCT study in accordance with the cystoscopic picture. The indications for biopsy were the following: suspicion of malignancy in the chronic inflammation group (12 specimens from 12 patients out of 67); suspicion of secondary damage (including the hemorrhage cases) or the presence of hemorrhage in the radiation damage group (9 specimens from 9 patients out of 105).
4. Preparation of histological sections.
5. TPM study of dewaxed unstained bladder tissue sections.
6. AFM study of the same histological sections.

To verify the results of SHG imaging, the same tissue samples underwent a standard histological evaluation with hematoxylin-eosin staining.

### 2.2. CP OCT Study

The first stage of the entire work was an in vivo study of the bladder by the CP OCT technique via the procedure described in [27]. The study was conducted using an “OCT 1300-U” cross-polarization optical coherence tomograph “OCT 1330-U” (BioMedTech Ltd., Nizhny Novgorod, Russia) certified for clinical application. The working wavelength of the device is 1300 nm (with a superluminescent diode as a source), power at the object is 3 mW, depth resolution is 20 µm, lateral resolution is 25 µm, and the rate of image acquisition is 200 A-scans/s. To obtain CP OCT images, an en-face endoscopic probe with the outer diameter of 2.7 mm was used.

Two conjugated images in co- and cross-polarizations are constructed. For the analysis, only image in cross-polarization is used, as the cross-polarization mode has higher specificity for collagen evaluation in comparison to the co-polarization mode [29,30]. The size of the OCT image in each polarization is 1.8 × 1.3 mm or 400 × 256 pixels (width × height).

**Figure 1:**
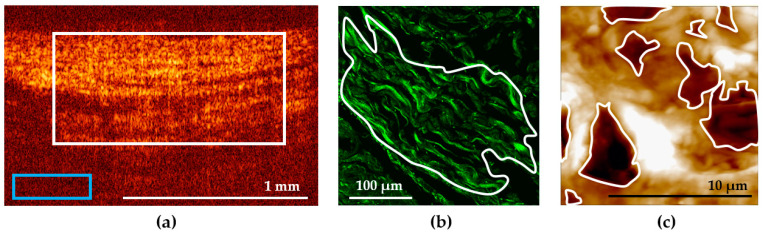
Examples of the ROI selection in the bladder images: (**a**) in a cross-polarization OCT image; (**b**) in an SHG image; (**c**) in an AFM image. ROI is marked with a white contour, the blue rectangle in (**a**) marks the background region selection.

The quantitative processing of cross-polarization images was performed using the ImageJ freeware (National Institutes of Health, Bethesda, MD, USA). Numerical analysis was performed for each of the 5 groups: “intact bladder” (34 images), “chronic cystitis” (107 images), “radiation cystitis Grade 2” (63 images), “radiation cystitis Grade 3” (56 images), and “radiation cystitis Grade 4” (46 images). For each considered cross-polarization image (Figure 1a), the average brightness of the OCT signal from an ECM region was calculated using the following procedure.

First, a region of interest (ROI) in the image was selected within the area of the bladder ECM. Note that the upper border corresponded to the border between the epithelium and the underlying connective tissue base, the lower border corresponded to the transition of the visible OCT signal to the noise, the left and right borders were selected so that the selected area occupied 50–75% of the image width and was located in the center (Figure 1a, white rectangle). The average level of the OCT signal was calculated from this region. The average level of the background signal was measured within the same image (Figure 1a, blue rectangle) and subtracted from the average level of the OCT signal calculated for the ROI. Then, the mean values (M) and standard deviations (SD) were calculated for each group, the values being presented as M ± SD.

### 2.3. TPM Study

TPM study of the connective tissue ECM harvested by biopsy was performed with TPM in the SHG mode using unstained dewaxed histological sections with a thickness of 10 µm, according to the procedure described in [15]. We used an LSM 510 META laser scanning microscopy system (Carl Zeiss, Jena, Germany). For excitation, we used a Ti:Sa femtosecond laser Mai Tai HP (Spectra Physics, CA, USA) with the pulse frequency of 80 MHz and pulse duration of 100 fs with a chosen wavelength of 800 nm. The average laser radiation power in the sample plane was less than 4 mW. Detection was performed using a filter in the range of 362–415 nm (SHG signal of collagen, green color in the images). Samples were placed on a 170 μm-thick coverslip. SHG imaging of collagen was performed in the reflective mode using an EC Plan-Neofluar oil-immersion objective (Carl Zeiss, Jena, Germany) with a 40× magnification and a numerical aperture of 1.3 that allowed us to obtain a 318 × 318 µm (1024 × 1024 pixels) field of view.

Quantitative estimation of the signal in SHG images was performed with the ImageJ freeware (National Institutes of Health, Bethesda, MD, USA) [16]. One to three ROIs were selected in the bladder wall images having a typical tissue structure (Figure 1b). The regions were contoured according to the following quality criteria: clear structure of collagen fibers, absence of out-of-focus dark fields, as well as absence of blood vessels, epithelium, and muscular fibers within the ROI. As a numerical criterion for the collagen state evaluation, a mean value of the SHG signal over a selected ROI was used. Then, we calculated the mean intensity of the SHG signal over all the ROIs for each dose and time post-irradiation.

### 2.4. AFM Study

AFM study was performed using 10 µm thick unstained dewaxed sections of the bladder tissue. The sections on glass slides were studied on air, in the semi-contact mode. Topography and phase images were acquired using a Solver P47 atomic force microscope (NT-MDT, Zelenograd, Russia), with a Solver scanner and TESP probes (Bruker, Santa Barbara, CA, USA) with a nominal spring constant of 42 N/m, nominal resonant frequency of 320 kHz, and a nominal tip radius of 8 nm. AFM images were acquired with a scan rate of 1 Hz and a resolution of 512 × 512 pixels. General view images measuring 14 × 14 µm of the ECM were obtained from different tissue parts, based on the corresponding images in the optical microscope combined with AFM. Furthermore, to demonstrate nude collagen fibrils in pathological conditions, we obtained AFM phase images with a high resolution (3 × 3 µm). To study the tissue morphology by AFM, images from no fewer than 10 different places were acquired. Qualitative estimation of AFM images involved characterization of the visual appearance of the structure and thickness of fibers and the presence and amount of the amorphous substance at the section surface. A quantitative description of AFM images of the ECM represents a predicament due to the random pattern of the ECM structural components. Thus, it is rarely ever applied in such studies. We made an attempt to quantitatively evaluate the disordering of the ECM in pathological conditions using the size of formed ruptures in the collagen network as a marker of the ECM deterioration. We calculated the ratio of the total area of deep (>1 µm) ruptures (holes) to the whole AFM image area (14 × 14 µm). The area calculations were performed using the ImageJ freeware (National Institutes of Health, Bethesda, MD, USA). Using the threshold function, we manually determined a threshold value of the vertical coordinate, below which a deep rupture relative to the mean topography level was located. Using the analyze function, we calculated the area inside the contoured regions formed by applying the threshold function (Figure 1c), and the fraction of the area occupied by such regions relative to the whole AFM image area. The second parameter estimated in AFM images was the D-period, the characteristic axial periodicity of fibrillary collagen (types I, 2, 2I, V and XI). The D-period is approximately 67 nm, and it varies depending upon the hydration state of the aggregate [31].

### 2.5. Statistical Processing

Statistical analysis was performed with the use of the Statistica 10.0 and SPSS Statistics 27 package. The sample belonging to the normal distribution was determined using the Shapiro–Wilk test. In each group, the values of the cross-polarization OCT signal, SHG signal, and the relative area of ruptures in AFM images were characterized by the mean and standard deviation (M ± SD). To compare the data for two independent groups, Student’s *t*-test was applied. The comparison of the quantitative characteristics between the selected groups was performed depending on their pathological state (the degree of radiation damage, chronic cystitis). Such a comparison facilitated the detection of radiation-induced changes of collagen and related them to the severity of the clinical manifestations of the bladder damage. The corresponding quantitative characteristics for the images of the intact bladder tissue were taken as the control. Differences were rendered statistically significant at *p* ≤ 0.05.

## 3. Results

### 3.1. The Study of the ECM at the Level of the General Organ Architecture by CP OCT

**Figure 2:**
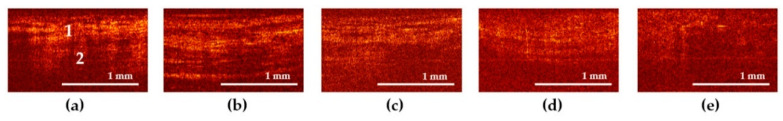
Typical cross-polarization OCT images of the bladder mucosa in the studied patients’ groups: (**a**) “normal” group; (**b**) “chronic cystitis” group; (**c**) “Grade 2 radiation cystitis” group; (**d**) “Grade 3 radiation cystitis” group; (**e**) “Grade 4 radiation cystitis” group. 1—mucosa and submucosa layers, 2—muscular layer.

Typical cross-polarization OCT images of the bladder mucosa for each of the analyzed groups are presented in Figure 2. The signal in the cross-polarization channel is generated by collagen fibers, which are present predominantly in the mucosa and submucosa (Figure 2a, number 1) and also from the strata between the muscles in the muscular layer (Figure 2a, number 2). The images of the normal tissue are characterized by a high-intensity inhomogeneous signal with a crisp layered structure. This allows for accurate differentiation of subepithelial connective tissue structures (the lamina propria of the mucosa and the submucosa are not resolved separately) (Figure 2a, zone 1) and the muscular layer of the bladder wall (Figure 2a, zone 2).

**Figure 3:**
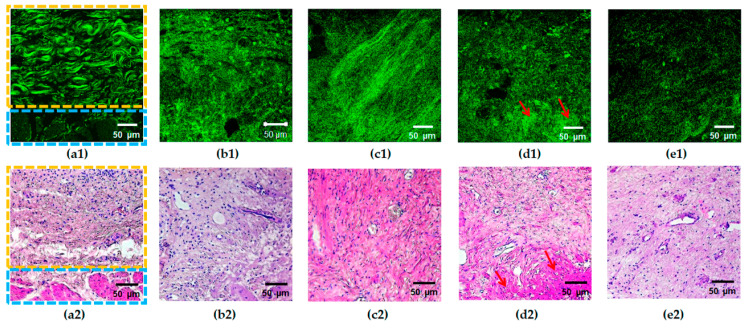
Typical SHG images (**a1**–**e1**) of the bladder mucosa with the corresponding histology (**a2**–**e2**) in the studied patients’ groups: (**a**) “normal” group, the border of the submucosal (marked with a yellow rectangle) and the muscular (marked with a blue rectangle) layers are clearly seen; (**b**) “chronic cystitis” group; (**c**) “Grade 2 radiation cystitis” group; (**d**) “Grade 3 radiation cystitis” group, hyalinosis structures are marked with red arrows; (**e**) “Grade 4 radiation cystitis” group.

In cross-polarization OCT images of the bladder with chronic cystitis, an expansion of the region with a high signal level is observed (Figure 2b), which we assign to thickening of the subepithelial structures as a result of the connective tissue proliferation, edema, and inflammation (Figure 3b2). In addition, regions with almost no signal appear, which are assumed to correspond to the locations of the interstitial fluid. The images retain the layered structure, as a rule.

Cross-polarization OCT images of the bladder with Grade 2 radiation cystitis (Figure 2c) are similar to those for chronic inflammation (Figure 2b); however, no significant thickening of the submucosa is observed, the average intensity level is lower, and the area of the no-signal regions is increased. With a further increase in the radiation damage degree (Figure 2d,e), the intensity of the cross-polarization signal decreases. Thus, the images lose their layered structure, and the signal becomes homogeneous (partially for Grade 3, completely for Grade 4). This finding is confirmed histologically by essential destruction of collagen fibers; while for Grade 3, one may still observe regions of collagen fibers with the preserved structure (Figure 3d2), and for Grade 4, massive destruction of the majority of fibers is registered (Figure 3e2) along with the pronounced tissue edema.

### 3.2. The Study of the ECM at the Level of Collagen Fibers and Their Bundles by TPM

In TPM images of the normal tissue in the SHG mode, the collagen structures are detected in the form of unidirectionally oriented wavy bundles, as shown in Figure 3a.

In the case of pathological changes, a characteristic feature of SHG images is a loss of the ordered structure and the presence of disturbances in the spatial arrangement of collagen fibers (Figure 3b–e). The latter manifests itself as the absence of the crisp contour of fibers (fuzziness) in a part of images (Figure 3b), disorder/fragmentation (Figure 3b–e), appearance of regions of dense disordered packing of fibers (Figure 3b–e), or structureless regions with a high SHG signal (Figure 3d). Previously, we demonstrated that for the bladder pathological conditions under study, the degree of the changes in collagen and elastic fibers visually increases from Grade 2 to 4 of radiation cystitis (Figure 3c–e), while the picture for chronic cystitis (Figure 3b) is similar to that of the Grade 2 radiation cystitis (Figure 3c) [28].

### 3.3. The Study of the ECM at the Level of Collagen Fibers by AFM

**Figure 4:**
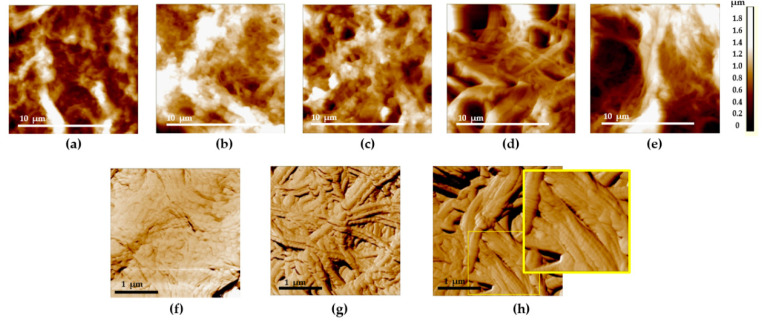
Typical AFM images of the bladder mucosa in the studied patients’ groups: (**a**,**f**) “normal” group; (**b**) “chronic cystitis” group; (**c**,**g**) “Grade 2 radiation cystitis” group; (**d**) “Grade 3 radiation cystitis” group; (**e**,**h**) “Grade 4 radiation cystitis” group. (**a**–**e**) 14 × 14 µm, topography: an increase in the deep ruptures’ area is seen from the left to the right. (**f**–**h**) 3 × 3 µm, phase: an increase in the area of denuded collagen fibrils is seen from the left to the right: full coverage of collagen fibrils with the amorphous substance (**f**), collagen fibrils are partially (**g**) and completely (**h**) denuded with the discernible D-period (see the enlarged view of the (**h**) image indicated by the yellow rectangle).

In the normal bladder ECM, collagen fibers are arranged in a peculiar wavy pattern, with densely interlaced fiber bundles. The packing pattern of collagen fibrils at the nanoscale generally repeats that of collagen fibers observed at the microscale (Figure 4a), and the fibrils are covered with an unstructured proteinaceous material (Figure 4f).

Such a pattern was present in the animal bladder ECM [25], as well as in certain other types of connective tissue (a “basket-weave” packing of collagen fibers in the skin or arterial walls) [19,32].

With the development of radiation cystitis, regions with loosened collagen network in the ECM appear at all the scales. In 3 × 3 µm images, along with the regions of fibrils covered with an unstructured proteinaceous material, nude collagen fibrils appear (Figure 4g), up to the complete loss of proteinaceous coverage and exposure of fibrils (Figure 4h). The main signs of the radiation damage of the bladder ECM involved the loosening of collagen fibrils’ packing and thickening and disordering of collagen fibrils. AFM data revealed that the ordered 3D network of the ECM collagen structures was almost entirely destroyed. The observed ECM rearrangement indicated not only the direct destruction of the ECM protein backbone, but also the onset of fibrotic changes (wide oriented collagen fibers). Thus, the AFM visualization of histological specimens revealed the destruction and remodeling of ECM in the bladder with radiation cystitis, similar to the features found earlier in animal experimental models [25].

### 3.4. Quantitative Evaluation of the Changes in the ECM Depending on the Level of Its Hierarchical Organization

The results of the quantitative analysis of CP OCT images, SHG and AFM images reflecting the degree of damage of the bladder ECM in the studied groups versus normal tissue are presented in Table 2.

For normal tissue, the average brightness of the OCT signal in cross-polarization was 37.1 ± 5.9 a.u., while for chronic cystitis, it was 25.6 ± 5.2 a.u. For Grade 2 radiation cystitis, the brightness of the OCT signal in cross-polarization images was 21.6 ± 5.9 a.u. For Grade 3 and 4, it was 20.1 ± 7.7 and 17.9 ± 5.5 a.u., respectively (Table 2). Such a noticeable drop in the OCT signal relative to the normal values indicate degradation of collagen-containing structures in the case of severe radiation cystitis. All the differences in pathology were statistically significant in comparison to the normal state at *p* ≤ 0.05.

The results of the quantitative analysis of SHG images showed that the average intensity of the SHG signal for the normal bladder tissue was 117.1 ± 20.0 a.u. For chronic cystitis, this parameter was 100.8 ± 16.0 a.u. For Grade 2 radiation cystitis, the average value of the SHG signal intensity (124.8 ± 21.0 a.u.) did not differ from that of the intact bladder. For Grade 3 and 4 radiation cystitis, this parameter was 87.6 ± 10.3 and 81.3 ± 3.2 a.u., respectively (Table 2). Such a decline in the values reflect marked disordering (fragmentation) of fibers and the loss of the normal quasi-crystalline structure of collagen. The latter leads to a significant decline in the collagen’s ability to generate a nonlinear response.

The representation of the CP OCT and TPM study results in the form of a correlation cloud and subsequent analysis revealed a moderate positive correlation (with the linear Pearson’s correlation coefficient of 0.58, at the significance level of *p* < 0.05) between the brightness of the OCT signal in cross-polarization and intensity of the SHG signal from the bladder wall.

The calculations of the overall area of deep ruptures (holes) in AFM images have demonstrated that there exists a clearly expressed dependency of this index on the degree of the ECM radiation damage (Table 2). The area occupied by ruptures almost linearly increases with the degree of the radiation damage severity, reaching the maximum for the Grade 4 radiation cystitis. The statistically significant differences with the intact bladder are observed for any degree of radiation cystitis severity, as well as between the Grades 2 and 4 of radiation cystitis.

## 4. Discussion

Discerning the intact structure of the bladder ECM prior to RT, as well as its radiation-induced alterations, is important for the timely correction of the adverse effects of radiation and preservation of the organ’s functional reserve. For many years, the early changes in the connective tissue after ionizing radiation were considered a part of the process of degradation and destruction of collagen structures resulting from the cytokine boost in the irradiated region [10]. The activation of the excessive collagen synthesis due to vascular damage and ischemia following RT is considered to be a basic pathogenetic mechanism for the development of the radiation adverse effects [13]. The molecular and cellular mechanisms of the radiation damage development and its consequences for normal tissues have been studied extensively in the literature. However, the processes of collagen destruction and its subsequent remodeling at different levels of its hierarchical organization (fibers, fiber bundles, general ECM architecture) appears to have been largely overlooked. The use of optical and physical techniques for tissue studies has allowed for the previously unachievable estimation of the bladder collagen state at all the levels of its hierarchical organization after the action of clinically relevant doses of ionizing radiation.

The novel use of AFM in a study of the stated nature unearthed the correlation between ECM changes and the degree of the radiation damage severity. Although AFM is extensively applied in the studies on the ECM of different tissues, no systematic research utilizing AFM has been conducted so far on the radiation-affected human bladder ECM. In our previous study using an animal model [25], we demonstrated dependence of the early changes in the bladder ECM structure on the radiation dose. Here, we implement this approach to the clinical samples from patients after radiation therapy in addition to a novel quantitative assessment of the ECM structural alterations.

We have demonstrated that a substrate of the late radiation changes of the organ at the level of fibrils and fibers is represented by the following factors:

1. Disappearance of the ordered densely packed 3D network of collagen fibers, disordering of the ECM structure.
2. Appearance of a large amount of a structureless material covering the fibrous collagen structures in the case of Grade 2 radiation damage. For Grade 3–4 radiation damage, appearance of “nude” fibers not covered with the amorphous substance becomes more characteristic (Figure 4d,e).
3. Direct destruction of the bladder ECM, which is noticed in AFM images with the appearance of >1 µm-deep ruptures in the network of collagen fibers.

It is worthwhile noting that as the degree of radiation cystitis severity increases, the processes of the ECM destruction begin to prevail over neocollagenesis. This is displayed by the increase in the total area of ruptures in AFM images. The overall area of such ruptures linearly increases with the degree of radiation cystitis severity. We speculate that this is likely the origin of the reduction in the bladder tissue strength and the development of hemorrhages and fistulae in Grade 4 radiation cystitis. Long-term chronic cystitis of bacterial etiology also leads to the remodeling of the bladder connective tissue. However, it is much less pronounced than that in radiation cystitis (Figure 4b).

The numerical processing of TPM images of the bladder ECM acquired in the SHG mode has demonstrated an inverse relationship between the SHG signal intensity and the degree of radiation cystitis severity (Table 2). The decrease in collagen nonlinear response intensity is a universal sign of degradation and disordering of collagen structures at the level of fibers and fiber bundles in various pathological processes [33]. It should be noted that chronic cystitis also leads to disordering of the ECM that is reflected by the signal intensity decline in SHG images (Table 2).

CP OCT is a technique, which allows us to obtain an integral in vivo assessment of the ECM state at the level of the general architectonics of an organ [29,30] based on the polarization properties of its components. This study has shown that the decline in the OCT signal brightness in cross-polarization images from the bladder wall is a typical sign of radiation damage. The magnitude of decline appears to be correlated with the severity of the complication. The processes of the matrix degradation and remodeling at the microscopic level could form a basis for the diagnosis of this phenomenon. The decrease in the OCT signal in cross-polarization images is a universal sign of ECM disordering, which is noted in different pathological conditions of the tissue, including inflammation and malignancy [27,32,34]. The obtained results demonstrate that CP OCT adequately reflects the microstructural processes in the bladder tissue and may become an instrument for non-invasive monitoring of radiation-induced lesions in clinical practice.

## 5. Conclusions

The observed correspondence between ECM changes at the microscopic level and at the level of the general organ architectonics is suggestive that CP OCT could be considered as a viable method of “optical biopsy” in the grading of radiation-induced collagen damage.

## Figures and Tables

**Table 1:** Data of the patients included in the study.

Group	Patients without Pathology of the Bladder	Patients with Chronic Cystitis	Patients after RT: The Radiation Cystitis Severity Grades 2/3/4
(a) Total number of patients	20	67	105: 42/35/28
Age range (mean), years, for (a)	44–67 (52)	32–64 (53)	35–80 (56)
(b) Number of patients enrolled in CP OCT, TPM and AFM studies	5	12	9:3/3/3
Age range (mean), years, for (b)	50–65 (57)	38–55 (43)	37–79 (55)

**Table 2:** Results of the quantitative analysis of CP OCT images, SHG and AFM images reflecting the degree of damage of the bladder ECM.

Group/Calculated Parameter	“Norm”	“Chronic Cystitis”	“Grade 2 Radiation Cystitis”	“Grade 3 Radiation Cystitis”	“Grade 4 Radiation Cystitis”
Brightness of the OCT signal in cross-polarization image, a.u.	37.1 ± 5.9	25.6 ± 5.2 ^0^	21.6 ± 5.9 ^0^	20.1 ± 7.7 ^0^	17.9 ± 5.5 ^0^
Intensity of the SGH signal, a.u.	117.1 ± 20.0	100.8 ± 16.0	124.8 ± 21.0 *	87.6 ± 10.3 ^#,0^	81.3 ± 3.2 ^#,0^
Area occupied by ruptures in AFM images, %	7.8 ± 1.8	9.7 ± 2.5	17.8 ± 5.2 *^,#,0^	19.7 ± 5.0 *^,#,0^	24.8 ± 4.0 ^#,0^

Statistically significant differences: ^0^—between the groups with pathology and normal tissue, *p* ≤ 0.05; *—between the groups of Grade 2 and Grade 3–4 radiation cystitis, *p* ≤ 0.05; ^#^—between the groups of radiation and chronic cystitis, *p* ≤ 0.05.
